# Setrusumab for the treatment of osteogenesis imperfecta: 12-month results from the phase 2b asteroid study

**DOI:** 10.1093/jbmr/zjae112

**Published:** 2024-07-16

**Authors:** Francis H Glorieux, Bente Langdahl, Roland Chapurlat, Suzanne Jan De Beur, Vernon Reid Sutton, Kenneth E S Poole, Kathryn M Dahir, Eric S Orwoll, Bettina M Willie, Nicholas Mikolajewicz, Elizabeth Zimmermann, Seyedmahdi Hosseinitabatabaei, Michael S Ominsky, Chris Saville, James Clancy, Alastair MacKinnon, Arun Mistry, Muhammad K Javaid

**Affiliations:** Departments of Surgery, Pediatrics and Human Genetics, Shriners Hospitals for Children, McGill University, Montreal, Quebec H4A 0A9, Canada; Department of Endocrinology and Internal Medicine, Aarhus University Hospital, Aarhus, Middle Jutland 8200, Denmark; Department of Clinical Medicine, Aarhus University, Aarhus, Middle Jutland 8200, Denmark; Inserm UMR 1033, Edouard Herriot Hospital, 69372 Lyon cedex 08, France; Department of Medicine, Johns Hopkins University School of Medicine, Baltimore, MD 21287, United States; Department of Molecular & Human Genetics, Baylor College of Medicine & Texas Children’s Hospital, Houston, TX 77030, United States; Department of Medicine & Cambridge NIHR Biomedical Research Centre, University of Cambridge, Cambridge CB3 0FA, United Kingdom; Division of Endocrinology, Vanderbilt University Medical Center, Nashville, TN 37232, United States; Division of Endocrinology, Diabetes and Clinical Nutrition, School of Medicine, Oregon Health & Sciences University, Portland, OR 97239, United States; Faculty of Dental Medicine and Oral Health Sciences, McGill University, Montreal H3A 2T5, Canada; Shriners Hospitals for Children, Montreal, Quebec H4A 0A9, Canada; Faculty of Dental Medicine and Oral Health Sciences, McGill University, Montreal H3A 2T5, Canada; Shriners Hospitals for Children, Montreal, Quebec H4A 0A9, Canada; Faculty of Dental Medicine and Oral Health Sciences, McGill University, Montreal H3A 2T5, Canada; Shriners Hospitals for Children, Montreal, Quebec H4A 0A9, Canada; Faculty of Dental Medicine and Oral Health Sciences, McGill University, Montreal H3A 2T5, Canada; Shriners Hospitals for Children, Montreal, Quebec H4A 0A9, Canada; Ultragenyx Pharmaceutical Inc., Novato, CA 94949, United States; ICON Plc, Leopardstown, Dublin D 18, Ireland; Mereo BioPharma, London W16 0QF, United Kingdom; Mereo BioPharma, London W16 0QF, United Kingdom; Mereo BioPharma, London W16 0QF, United Kingdom; Nuffield Department of Orthopaedics, Rheumatology and Musculoskeletal Sciences (NDORMS), University of Oxford, Wellington Square, Oxford OX1 2JD, United Kingdom

**Keywords:** bone mineral density, osteogenesis imperfecta, setrusumab, sclerostin, rare disease

## Abstract

Osteogenesis imperfecta (OI) is a rare genetic disorder commonly caused by variants of the type I collagen genes *COL1A1* and *COL1A2*. OI is associated with increased bone fragility, bone deformities, bone pain, and reduced growth. Setrusumab, a neutralizing antibody to sclerostin, increased areal bone mineral density (aBMD) in a 21-week phase 2a dose escalation study. The phase 2b Asteroid (NCT03118570) study evaluated the efficacy and safety of setrusumab in adults. Adults with a clinical diagnosis of OI type I, III, or IV, a pathogenic variant in *COL1A1/A2*, and a recent fragility fracture were randomized 1:1:1:1 to receive 2, 8, or 20 mg/kg setrusumab doses or placebo by monthly intravenous infusion during a 12-mo treatment period. Participants initially randomized to the placebo group were subsequently reassigned to receive setrusumab 20 mg/kg open label. Therefore, only results from the 2, 8, and 20 mg/kg double-blind groups are presented herein. The primary endpoint of Asteroid was change in distal radial trabecular volumetric bone mineral density (vBMD) from baseline at month 12, supported by changes in high-resolution peripheral quantitative computed tomography micro-finite element (microFE)-derived bone strength. A total of 110 adults were enrolled with similar baseline characteristics across treatment groups. At 12 mo, there was a significant increase in mean (SE) failure load in the 20 mg/kg group (3.17% [1.26%]) and stiffness in the 8 (3.06% [1.70%]) and 20 mg/kg (3.19% [1.29%]) groups from baseline. There were no changes in radial trabecula vBMD (*p*>05). Gains in failure load and stiffness were similar across OI types. There were no significant differences in annualized fracture rates between doses. Two adults in the 20 mg/kg group experienced related serious adverse reactions. Asteroid demonstrated a beneficial effect of setrusumab on estimates of bone strength across the different types of OI and provides the basis for additional phase 3 evaluation.

## Introduction

Osteogenesis imperfecta (OI) is a rare genetic disorder of connective tissue associated with reductions in bone mass and increased bone fragility.^1^ Numerous OI types are classified by associated clinical and genetic characteristics, the majority (85%–90%) of which are caused by variants in the type I collagen genes *COL1A1* and *COL1A2.*^2^ Pathogenic variants can result in type I collagen haploinsufficiency, often associated with a lower disease burden, or structurally abnormal type I collagen, which presents with higher disease burden.^1^ In the latter, protein sequence changes including missense or splice site variants may result in altered protein composition, collagen chain misalignment, or disruption of the C-propeptide leading to misfolding, all of which contribute to structural abnormalities.^1^ This can result in overprocessing in the endoplasmic reticulum, protein misfolding, and ultimately lead to extracellular matrix dysfunction.^1^^,^^3^ The added cellular stress of extensive posttranslational modification can induce autophagy, apoptosis, and impair osteoblast differentiation, further reducing bone formation and exacerbating disease presentation.^1^

Pathogenic variants associated with OI outside of *COL1A1* and *COL1A2* play other roles in the type I collagen pathways, including disruption of bone mineralization, posttranslational modification, processing, and crosslinking of collagen, or osteoblast differentiation and function.^1^ Together, these variants comprise 4 clinical OI types (I–V), which exhibit various presentations and severity.^2^^,^^4^ Most commonly, OI type I is associated with quantitative mutations in which one or more collagen alleles are null, while OI types III/IV are associated with qualitative mutations including substitutions and splice site mutations.^5^^,^^6^ The overall prevalence of OI, as estimated from 2 European population-based studies, is between 1:9700 and 13 500 people^5^^,^^7^^,^^8^ with an individual prevalence of 1:19 400, 112 400, and 74 100 for OI types I, III, and IV, respectively.^5^ OI is characterized by increased rates of long bone and vertebral fractures, bone deformities, bone pain, and reduced growth.^1^^,^^2^ Although variable between individuals, the rates of fracture in OI generally follow a bimodal distribution. By far, the highest fracture rate is during childhood; however, fracture rates remain elevated throughout adulthood, especially after age 50.^9^ Additional manifestations of OI include joint laxity, hearing loss, muscle weakness, pulmonary compromise, and fatigue.^1^^,^^10^^,^^11^ Together, the high incidence of fractures and related clinical features impose a substantial disease burden and negative impact on the daily lives of both patients with OI and caregivers. Beyond regular health care visits, the increased fracture rate associated with OI requires frequent hospital visits, with fear and uncertainty of when the next fracture will occur commonly reported as impacting daily life.^12^ Additionally, costly medical appointments, physical and occupational therapies, medical devices, and a limited ability to work can all contribute to the substantial financial burden of OI.^12^

In OI, pathogenic *COL1A1* and *COL1A2* variants contribute to increased bone fragility in part through reductions in bone matrix strength stemming from alterations in collagen resulting in an increased mineral-matrix ratio.^13^^,^^14^ In addition, these variants result in imbalanced bone turnover with greater resorption than formation leading to low bone mass and poor bone architecture that further contribute to increased bone fragility.^15^ High-resolution peripheral quantitative computed tomography (HR-pQCT) allows the measurement of bone microarchitecture and volumetric bone mineral density (vBMD) in patients with OI at the peripheral skeleton.^15^^,^^16^ In cross-sectional studies utilizing HR-pQCT and DXA), adults with OI show decreases in radial bone area, and altered bone architecture including decreased trabecular number, increased trabecular spacing, reduced trabecular homogeneity, as well as decreased areal BMD (aBMD) and vBMD compared with controls.^17-20^

An unmet need exists for an effective therapeutic to improve bone health and reduce fracture risk in adults with OI.^10^ Although bisphosphonates are often used off-label to treat children with OI, their effectiveness in adults is unclear.^10^^,^^18^ Most often, bisphosphonates are discontinued after cessation of growth, reducing the availability of treatment options for adults.^21^ Administration of the anabolic agent teriparatide (PTH 1–34) for 18 mo has led to significant increases in LS aBMD (+6.1%) from baseline and estimated bone strength by micro-finite element (FE) analysis (+15.2%) in adults with OI type I; however, it showed limited efficacy in patients with OI types III and IV.^22^ Together, these studies emphasize the unmet need for therapy that will benefit adults with OI and treat patients across OI subtypes.

Research has investigated the effects of antisclerostin antibodies (anti-Scl-Ab) in osteoporotic bone disorders. Sclerostin (*SOST)* is a small protein produced primarily by osteocytes that inhibits the canonical Wnt signaling pathway through binding to LRP5 and 6, suppressing osteoblast differentiation and bone formation and increasing osteocyte/osteoblast RANKL-mediated osteoclastogenesis and bone resorption.^23^ Loss of sclerostin in the genetic diseases sclerosteosis and van Buchem disease causes elevated BMD and reduces risk of fracture.^24^ Therapeutic inhibition of sclerostin (via monoclonal anti-Scl-Ab) increases BMD in patients with osteoporosis.^25^ Serum sclerostin levels are shown to be significantly lower in adults with OI types I and III/IV, suggesting a possible negative feedback mechanism to diminishing BMD.^26^^,^^27^ However, this measure of serum sclerostin does not reflect sclerostin levels within osteocytes, which remain unknown.^28^ In mouse models of OI, anti-Scl-Abs have led to increased bone formation and mass while reducing bone fragility at both trabecular and cortical sites.^29-32^ Collectively, these findings suggest that restoring the abnormal turnover pathway may improve bone strength and mass, opening new options for therapeutics.

Setrusumab is an investigational fully human neutralizing IgG2λ monoclonal anti-Scl-Ab, which holds potential as a novel treatment option for patients with OI. Although convention suggests that altered collagen structure and availability weaken the tensile strength of bones resulting in fragility, it is the innate response to weakened bone that must be addressed in patients with OI. Detection of abnormal bone leads to excess resorption leading to a net deficit in bone production, ultimately leading to thinner, less dense bones with less strength. Sclerostin inhibition decreases bone resorption and increases bone formation at sites of bone tension and fragility, where remodeling occurs naturally. Thus, bone mass is added at sites lacking in bone through reduction of excessive bone resorption and an increase bone formation, yielding a positive bone balance, unlike currently available treatments. In a 21-week, open-label, phase 2a dose-escalation trial of setrusumab in adults with OI (NCT01417091), a statistically significant increase in bone formation markers and LS aBMD occurred, while bone resorption markers decreased.^33^ In that study, participants receiving setrusumab through the highest dose of 20 mg/kg reported no adverse events attributed to treatment, and no treatment-related fractures were observed.^33^

The phase 2b Asteroid trial (NCT03118570) is a parallel-group, dose-finding study of 3 doses of setrusumab over a 10-fold dose range, which was conducted to determine the dose of setrusumab that best improved bone parameters after 12 mo of treatment.

## Materials and methods

### Study design

Asteroid was a phase 2b, multicenter, multinational, double-blind, dose range finding study that evaluated 3 doses of setrusumab in participants with OI types I, III, and IV from September 2017 through November 2020. Enrollment for Asteroid was conducted at 25 study centers in the United States, United Kingdom, France, Denmark, and Canada.

The study design of Asteroid is summarized in Figure S1. Eligible participants were initially randomized using an interactive web response system 1:1:1:1 to receive 1 of 3 setrusumab doses (2, 8, or 20 mg/kg) or placebo by monthly intravenous infusion during a 12-mo treatment period. At a median of 5 mo (range: 1–11 mo), 19 participants initially randomized to the placebo group were subsequently reassigned to receive setrusumab 20 mg/kg open label for 12 mo. Two participants who enrolled in the study after the reassignment received only setrusumab 20 mg/kg open label. Due to inconsistencies in duration of placebo prior to beginning 20 mg/kg open label therapy, only the randomized setrusumab treatment groups are included in this analysis. Subjects were required to receive concomitant therapy with active vitamin D and/or calcium supplements.

The study protocol was approved by the appropriate independent ethics committee or institutional review board for each center and complied with the ethical principles of the Declaration of Helsinki and good clinical practice. All participants provided written informed consent.

### Participants

Participants were eligible to participate in Asteroid if they had a clinical diagnosis of OI type I, III, or IV with a confirmed mutation in *COL1A1*/*COL1A2*, were 18–74 yr of age, and had experienced one or more nontraumatic long bone, rib, hand/foot, and/or vertebral fractures within the previous 2 yr (amended to within the previous 5 yr on May 18, 2018). The study was open to both male and female participants; female participants were eligible only if they were not pregnant, not breastfeeding, and were either not of child-bearing age or agreed to follow contraceptive guidance during the treatment period.

The exclusion criteria of Asteroid had a history of skeletal malignancies or bone metastases, foraminal stenosis not due to scoliosis, abnormal thyroid function, a separate skeletal condition that could contribute to long bone deformities or increased risk of fracture, external radiation therapy, or those who had received treatment with bisphosphonates within 3 mo of baseline visit, or anabolic and antiresorptive medication (including teriparatide or denosumab) within 6 mo of the baseline visit.

### Outcome measures

The primary endpoint in Asteroid was the change from baseline to 12 mo in trabecular vBMD of the radius using HR-pQCT. The radius was selected as the primary skeletal site over the tibia to reduce the potential contribution of weight bearing to therapeutic response across the participants with OI.^15^ Trabecular vBMD was selected as it is considered most responsive to anabolic therapy when compared with other HR-pQCT parameters.^34^ The primary endpoint was supported by microFE-based estimates of bone stiffness and failure load. These additional supportive endpoints were selected as stiffness, and failure load has been demonstrated to be independent predictors of fracture.^35^^,^^36^ Secondary endpoints included measures of changes in total and cortical vBMD of the radius and tibia, bone geometry and microarchitecture, stiffness and failure load of the tibia, aBMD, markers of bone turnover, rates of fracture, and safety outcomes.

#### High-resolution peripheral quantitative computed tomography

High-resolution peripheral quantitative computed tomography scans were performed on the nondominant distal radius and tibia at baseline and month 12 using a Scanco XtremeCT (*n* = 7 HR-pQCT scanning centers) or XtremeCT II (*n* = 6 HR-pQCT scanning centers) scanner (Scanco Medical AG). The dominant limb was scanned for cases in which the nondominant limb was supported with hardware present in volume of interest, had a recent fracture, or a significant deformity did not allow the limb to enter the HR-pQCT scanner. The methods, procedures, and trainings for HR-pQCT image acquisition and analysis were developed by a central HR-pQCT lab (Willie lab), and images were analyzed with readers blinded to treatment assignments and clinical information. Density and microarchitectural outcomes were reported from the common volumes of interest identified using cross-sectional area registration after month 12. Additional details are provided in the Supplementary Methodology.

#### MicroFE analysis

MicroFE was used to estimate bone stiffness, failure load, and apparent modulus of the radius and tibia from HR-pQCT images by the central HR-pQCT lab in a similarly blinded manner.^37^ Changes from baseline in radial stiffness and failure load were assessed at month 12. Other secondary microFE endpoints were changes from baseline in stiffness and failure load of the tibia at month 12. Additional details are provided in the Supplementary Methodology.

#### Dual-energy x-ray absorptiometry

DXA scans were performed at baseline and months 6 and 12 using the GE Lunar enCORE (Prodigy, Prodigy Advance, iDXA, and DPX-NT Series) and Hologic (QDR-4500, Delphi, Discovery, Horizon, and Explorer Series) scanners. For each patient, all DXA scans were performed on the same scanner. For each scanner, a baseline calibration reference point was established based on phantom data collected and the date of the first subject scan on that particular scanner. Additional details are provided in the Supplementary Methodology.

aBMD was evaluated in the LS (L1 to L4), total body excluding the head, TH, and FN, with changes from baseline at month 12 as secondary endpoints. Scans were read at the central imaging center (BioClinica, now Clario) blinded to treatment allocation and sequence but not the participant. Change in trabecular bone score (TBS) from baseline was assessed from DXA images using iNsight software. TBS is a gray-level measurement reflecting trabecular microarchitecture and has been shown to be predictive of vertebral and major osteoporotic fractures independent of BMD.^19^^,^^38^

#### Analysis of bone turnover biomarkers

Changes from baseline in serum biomarkers of bone turnover were assessed as additional secondary outcome measures. Fasting predose blood samples (10 mL) were collected at baseline and months 1, 3, 6, 9, and 12 for analysis of serum P1NP, osteocalcin (OC), bone-specific alkaline phosphatase (BSAP), and collagen cross-linked C-telopeptide type 1 (CTx1). Additional details are provided in the Supplementary Methodology.

#### Fractures

On-study fractures were reported by study participants and confirmed by radiograph at the central imaging center blinded to treatment allocation (BioClinica). Only reported nonvertebral fractures were examined—no imputations were made. Lateral spine radiographs were taken at baseline and month 12 and assessed for new morphometric vertebral fractures by Genant’s semiquantitative grading (GSQ) scale (grade 0: normal, <15% height loss; grade 1: mild fracture, 15%–25% height loss; grade 2: moderate fracture, 26%–40% height loss; grade 3: severe fracture, >40% height loss) blinded to treatment allocation.^39^ New vertebral fractures with GSQ grade of at least 1 post baseline were considered new fractures, and a change in GSQ grade of at least 1 from previous visit indicated a worsening fracture. Rates of new on-study fractures per participant year were calculated, and the number of fractures by location is reported descriptively for each treatment group.

#### Safety and tolerability

Safety and tolerability outcomes assessed the number of treatment emergent adverse events (TEAE), serious TEAEs, and infusion-related reactions reported during the course of the study using the Medical Dictionary for Regulatory Activities (MedDRA®, version 23.1; https://www.meddra.org/index.asp). MedDRA terminology is the international medical terminology developed under the auspices of the International Council for Harmonisation of Technical Requirements for Pharmaceuticals for Human Use (ICH). At specified time points during the study, predose serum samples were assessed for the presence of antidrug antibodies (ADA).

### Statistical analysis

Statistical methodology is available in the Supplementary Methodology.

## Results

### Baseline characteristics

In Asteroid, 110 enrolled participants were randomized to receive setrusumab at 2, 8, 20 mg/kg, or placebo once monthly by intravenous infusion for 12 mo. The placebo arm was replaced by a 20 mg/kg setrusumab open label group by protocol amendment, and after a mean of 5 mo, 19 of 20 participants in the placebo group were reassigned to receive 20 mg/kg setrusumab open label; one participant discontinued from the study before switching. Two additional participants were randomized into the open label group, resulting in total of 30, 29, 31, and 21 participants in the 2, 8, 20 mg/kg, and 20 mg/kg open label groups, respectively. Results from the setrusumab 20 mg/kg open label group were generally comparable to the 20 mg/kg randomized treatment group and are provided separately in the Table S1.

Baseline demographics and characteristics are summarized in Table 1. The mean age at baseline across randomized setrusumab groups was 42.7 yr (range: 19–74 yr). Most participants in the randomized setrusumab groups were female (64.4%), White (94.4%), and had a diagnosis of OI type I (61.1%). Baseline characteristics were generally similar across treatment groups, including height, weight, and body mass index. Notable between-group differences were participants who received setrusumab 2 mg/kg, had a higher mean age than the other groups, and a smaller proportion of participants in the setrusumab 20 mg/kg group were female. Prior use of bisphosphonates was infrequent in this adult population, ranging from 7% in the 8 mg/kg group to 19% in the 20 mg/kg group (Table 1). Baseline DXA T-scores and Z-scores are included separately in Table S2.

**Table 1:** Baseline demographics and characteristics.

**Parameter** **Statistics**	**Setrusumab dose**		
	**2 mg/kg (*n* = 30)**	**8 mg/kg (*n* = 29)**	**20 mg/kg (*n* = 31)**
**Age (years)**			
** Mean (SD)**	47.2 (12.4)	40.4 (14.3)	40.6 (13.7)
** Min, Max**	31, 74	20, 67	19, 73
**Sex, *n* (%)**			
** Female**	21 (70.0)	20 (69.0)	17 (54.8)
**Race, *n* (%)**			
** Black or African American**	1 (3.3)	1 (3.4)	2 (6.5)
** White**	29 (96.7)	27 (93.1)	29 (93.5)
** Not collected or reported**	0	1 (3.4)	0
**Ethnicity, *n* (%)**			
** White**	28 (93.3)	27 (93.1)	27 (87.1)
** Hispanic or Latino**	2 (6.7)	1 (3.4)	3 (9.7)
** Not reported or unknown**	0	1 (3.4)	1 (3.2)
**Height (cm)**			
** Mean (SD)**	150.7 (19.2)	150.6 (22.0)	150.4 (17.4)
** Min, Max**	93.5, 172.7	76.0, 180.9	119.0, 176.3
**Weight (kg)**			
** Mean (SD)**	64.8 (18.2)	65.1 (20.5)	63.2 (18.9)
** Min, Max**	34.7, 125.9	19.9, 120.7	37.4, 109.5
**BMI (kg/m** ^**2**^ **)**			
** Mean (SD)**	29.4 (8.7)	28.3 (5.0)	28.3 (10.5)
** Min, Max**	18.4, 52.5	21.5, 41.3	18.5, 76.2
**OI type at randomization, *n* (%)**			
** Type I**	19 (63.3)	18 (62.1)	18 (58.1)
** Type III/IV**	11 (36.7)	11 (37.9)	13 (41.9)
**Prior bisphosphonate use, *n* (%)**	4 (13.3)	2 (6.9)	6 (19.4)
** Zoledronic acid**	2 (6.7)	1 (3.4)	4 (12.9)
** Risedronate sodium**	1 (3.3)	1 (3.4)	2 (6.5)
** Alendronate sodium**	1 (3.3)	0	0
**Baseline highest vertebral fracture grade, *n* (%)**			
** Grade 1**	9 (30.0)	5 (17.2)	3 (9.7)
** Grade 2**	6 (20.0)	6 (20.7)	9 (29.0)
** Grade 3**	3 (10.0)	2 (6.9)	5 (16.1)

Abbreviations: BMI, body mass index; OI, osteogenesis imperfecta; SD, standard deviation.

*N* = number of participants in the safety population. *n* = number of participants in the specified category with nonmissing values.

### Primary outcomes

The primary endpoint of change in radial trabecular vBMD from baseline at month 12 did not reach statistical significance (Table 2). Across groups, baseline radial trabecular vBMD ranged from −0.45 to 279.01 mg HA/cm^3^, reflecting a wide range of trabecular density prior to enrollment. The negative trabecular vBMD is likely an artifact of the software calculation of bone density wherein the site is dominated by the presence of fatty marrow, which has a density of <0 mg HA/cm^3^ and is not atypical in the distal radius (Figure S2). At month 12, the range for percent change from baseline for radial trabecular vBMD was also wide, from −486.7% to 15.8% (Table 2). The −486.7% change in the 2 mg/kg group occurred in a participant with minimal trabecular vBMD at baseline (−0.45 mg HA/cm^3^), which reflected a small absolute reduction from baseline (−2.19 mg HA/cm^3^).

**Table 2:** Radial trabecular vBMD by HR-pQCT.

**Radial HR-pQCT trabecular vBMD**	**Setrusumab 2 mg/kg (*n* = 30)**	**Setrusumab 8 mg/kg (*n* = 29)**	**Setrusumab 20 mg/kg (*n* = 31)**
**Baseline**			
** Mean (SEM), mg HA/cm^3^**	110.2 (7.3)	112.0 (8.9)	127.1 (8.2)
** Range, mg HA/cm^3^**	−0.45^a^, 182.6	18.2, 212.7	32.9, 279.0
**Month 12**			
** Mean (SEM), mg HA/cm^3^**	110.4 (8.1)	111.4 (9.9)	121.9 (6.8)
** Range, mg HA/cm^3^**	33.0, 201.6	29.5, 209.0	−2.6, 186.4
**% change from baseline at month 12**			
** Mean (SEM)**	−20.0 (19.5)	−0.6 (1.1)	0.7 (0.9)
** Range, mg HA/cm^3^**	−486.7^a^, 7.8	−12.0, 9.2	−10.7, 15.8
** *p*-value**	0.404	0.485	0.644

a negative trabecular vBMD was detected in a few participants as a result of the mean density being dominated by the presence of fatty marrow, which has a density of <0 mg HA/cm^3^*.*

Abbreviations: HR-pQCT, high-resolution peripheral quantitative computed tomography; vBMD, volumetric bone mineral density; HA, hydroxyapatite.

**Figure 1:**
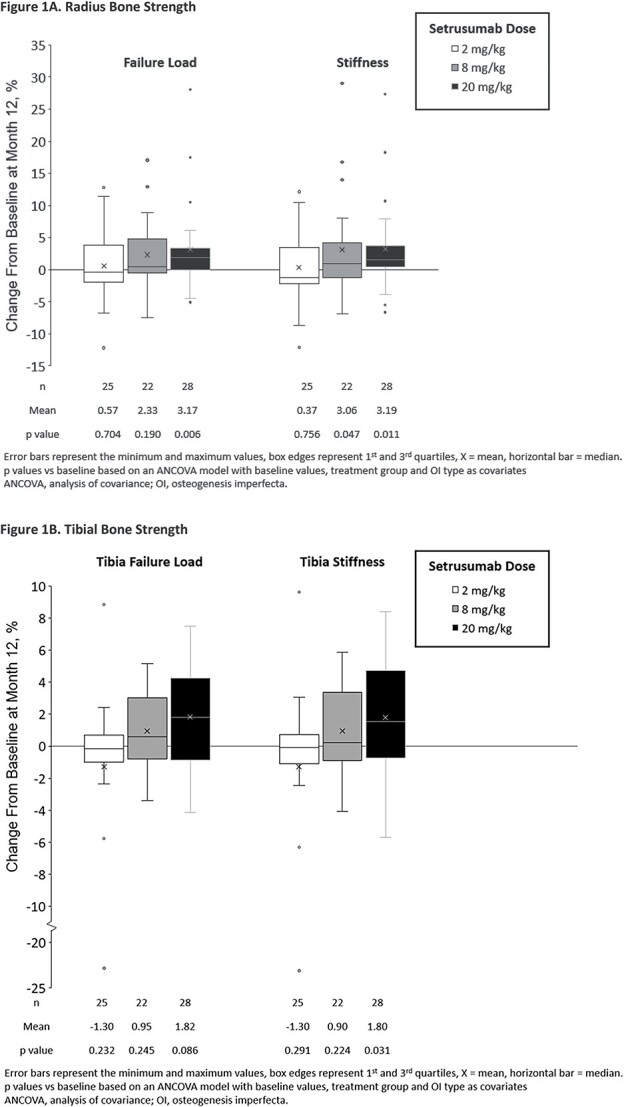
Changes in bone strength by microFE after 12 mo of setrusumab, A. Radius bone strength, B. Tibial bone strength.

Radial bone strength was assessed by microFE in support of the radial trabecular vBMD measure. This outcome demonstrated significant improvements from baseline to month 12 for failure load in the 20 mg/kg (*p*=.006) group and stiffness in the 8 mg/kg (*p*=.047) and 20 mg/kg (*p*=.011) groups (Figure 1A). Absolute change from baseline is included in Table S3.

### Secondary outcomes

#### High-resolution peripheral quantitative computed tomography

**Figure 2:**
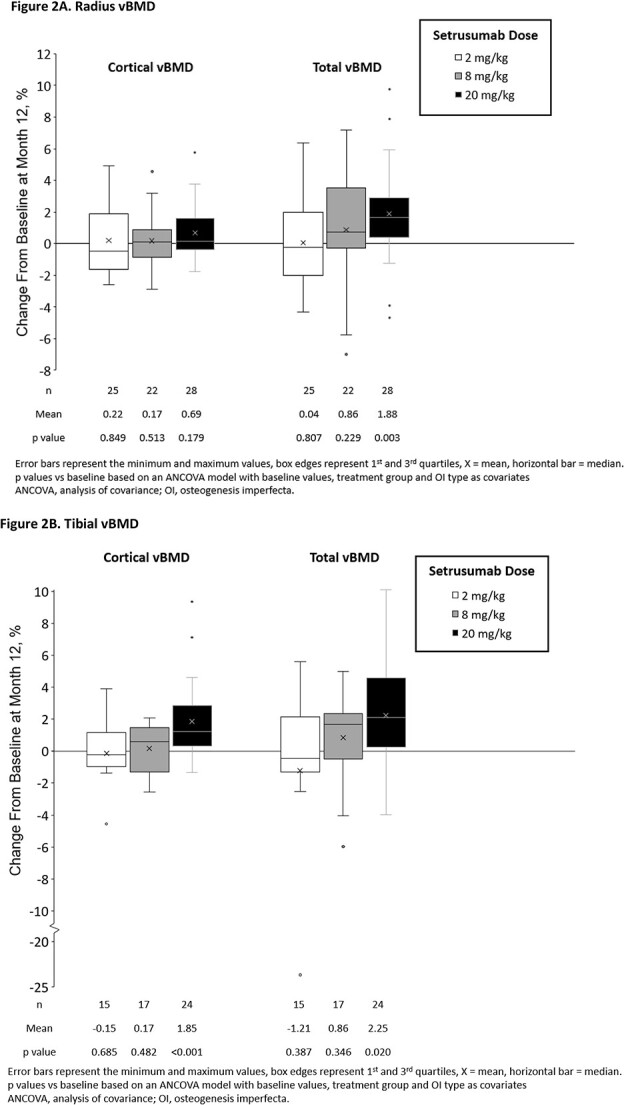
Changes in vBMD from baseline at month 12, A. Radius vBMD, B. Tibial vBMD.

Secondary HR-pQCT outcomes significantly improved with setrusumab treatment, including the percent change from baseline in cortical and total vBMD of the radius and tibia at month 12 (Figure 2). The total vBMD of the radius significantly increased in the 20 mg/kg group (*p*=.003) with no change in cortical vBMD (*p*=.179) (Figure 2A).

Similar to the data at the radius, trabecular vBMD of the tibia exhibited a broad range of values at baseline (range: 5.48–353.08 mg HA/cm^3^ across randomized setrusumab groups). A consistent effect of setrusumab was not observed at month 12 (mean [SEM] change from baseline: −2.6% [2.2%], 4.4% [7.2%], and 0.2% [1.3%] in the setrusumab 2, 8, and 20 mg/kg groups, respectively). Both cortical (*p*<.001) and total tibial (*p*=.02) vBMD increased significantly in the 20 mg/kg group (Figure 2B).

Consistent with its effects on trabecular vBMD, setrusumab did not result in significant changes in other trabecular microarchitecture endpoints at the radius or tibia at month 12. In contrast, in both the 8 and 20 mg/kg setrusumab groups, cortical area was significantly increased at both the radius (5.2%, *p*=.014 and 2.6%, *p*=.007, respectively) and tibia (2.6%, *p*=.024 and 3.9%, *p*=.039, respectively). Cortical thickness at the radius was also significantly increased (2.7%, *p*=.026 and 2.8%, *p*=.002, respectively) in these groups (Table S4).

Tibial stiffness increased significantly from baseline at month 12 in the 20 mg/kg group (*p*=.031); however, changes in failure load did not reach statistical significance (*p*=.086) (Figure 1B). Absolute changes from baseline are included in Table S3.

#### Dual-energy x-ray absorptiometry

**Figure 3:**
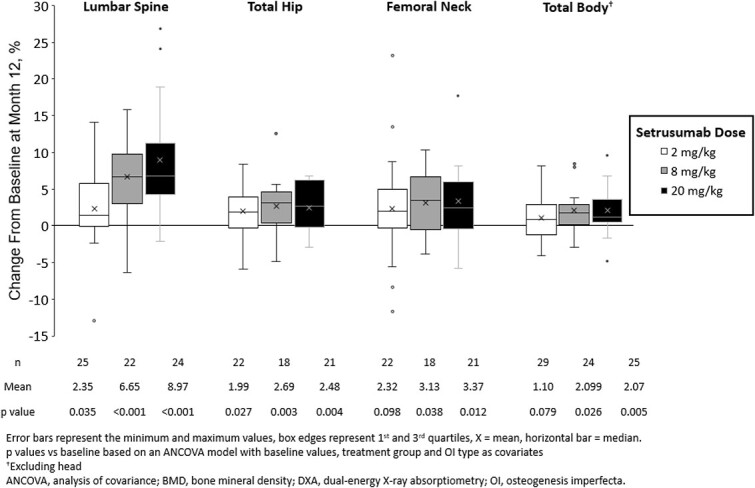
Areal bone mineral density by dual-energy x-ray absorptiometry scan.

DXA scans were available in 55 type I and 35 type III/IV OI participants. aBMD increased significantly from baseline to month 12 in all treatment doses at the LS (2 mg/kg: *p*=.035; 8 mg/kg and 20 mg/kg: *p*<.001) and TH (2 mg/kg: *p*=.027; 8 mg/kg: *p*=.003; 20 mg/kg: *p*=.004) (Fig. 3). The 8 and 20 mg/kg doses also significantly increased FN (*p*=.038 and .012, respectively) and total body aBMD (*p*=.026 and .005, respectively). Changes in aBMD over time, available in Figure S3A-D, demonstrated consistent improvements over the 12-mo treatment period.

Significant improvements from baseline to month 12 in TBS were observed in the 8 and 20 mg/kg setrusumab groups with mean percentage increases of 2.9% and 3.9%, respectively (both *p*<.001).

#### Bone biomarkers

**Figure 4:**
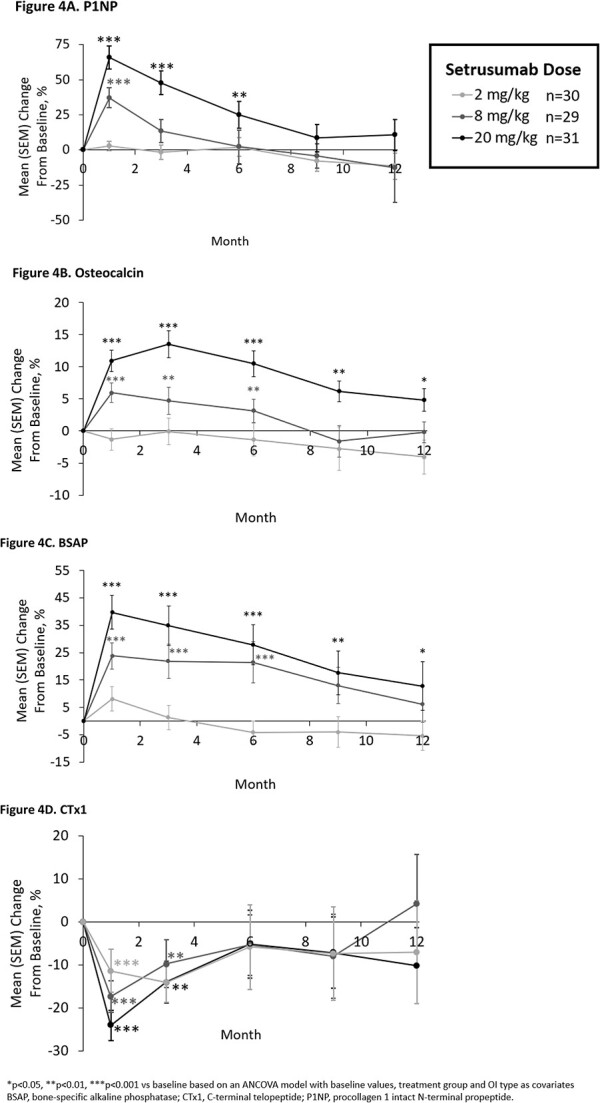
Changes in biomarkers of bone formation (A-C) and resorption (D) from baseline, A. Procollagen type 1 N-terminal propeptide, B. osteocalcin, C. bone-specific alkaline phosphatase, D. collagen cross-linked C-telopeptide type 1.

Biomarkers of bone formation significantly increased from baseline after treatment initiation in the 8 and 20 mg/kg groups, but not 2 mg/kg dose. Levels peaked at months 1–3 before gradually tapering toward baseline levels through month 12 (Figure 4A-C). CTx1, a marker of bone resorption, showed a similar but inverse trend with significant decreases from baseline at month 1 of setrusumab for all treatment doses, remaining significantly below baseline up to month 3 before gradually tapering toward baseline (Figure 4D).

Statistically significant (*p*<.001) positive correlations were observed between early changes in bone formation markers and later changes in LS aBMD across groups (Figure S4). No significant relationship was observed between the month 1 changes in the bone resorption marker CTx1 and month 12 changes in LS aBMD.

#### Analysis by OI type

Additional analyses examined the effects of setrusumab on key endpoints by OI type (I vs III/IV) (Figure S5A-D). No formal statistical testing was performed on these outcomes due to small sample sizes. However, in the 8 mg/kg and 20 mg/kg dose groups, both participants with OI types I and III/IV showed consistent numerical improvements from baseline in LS aBMD, radial failure load, serum P1NP, and serum CTx1 at month 12.

#### Fracture rate

Radiographically confirmed fractures that occurred during the 12-mo treatment period are shown in Table S5 for the setrusumab 2 (10 fractures in 7 participants), 8 (14 fractures in 10 participants), and 20 mg/kg (5 fractures in 5 participants) groups. The annualized fracture rates per participant year were 0.38, 0.54, and 0.19 for setrusumab 2, 8, and 20 mg/kg groups, respectively.

Clinical vertebral fractures were not reported for any participant in the randomized treatment groups. Two new morphometric vertebral fractures were observed at month 12, one each in the 8 and 20 mg/kg groups, and one worsening fracture was reported in the 8 mg/kg group. Notably, no significant changes from Baseline were observed in mean vertebral fracture grades in any group through month 12. The average sum of vertebral fracture grades at baseline was 3.7, 2.9, and 4.8 per participant in the 2, 8, and 20 mg/kg groups, respectively, highlighting the prevalence of vertebral morphology changes in this adult OI population. Notably, most fractures detected using lateral spine radiographs assessed using GSQ were asymptomatic and would not have been counted in those reported by participants.

### Safety outcomes

In the 12-mo treatment period, TEAEs were reported in 90%–100% of participants in the setrusumab treatment groups. The majority of these TEAEs were classified as mild or moderate. Four subjects (2 each in 20 mg/kg and 20 mg/kg open label groups) had TEAEs that led to study discontinuation (neutropenia or headache, headache, or worsening platybasia-related symptoms, respectively) (Table 3). Serious TEAEs were reported in 13.3%, 24.1%, 12.9%, and 23.8% of participants in the setrusumab 2, 8, 20 mg/kg, and 20 mg/kg open label groups, respectively (Table 4). Two participants in the setrusumab 20 mg/kg group (anaphylaxis in 1 participant; headache and hydrocephalus in 1 participant) and 2 participants in the 20 mg/kg open label group (headache and chills in 1 participant, pulmonary hypertension in 1 participant) had serious TEAEs considered to be related to treatment by the study investigator. Notably, no safety signals for neurologic impingement or skeletal events (excluding fractures) or major cardiovascular events were reported. Infusion site reactions during the 12-mo treatment period were reported in 3.3%, 6.9%, 9.7%, and 14.3% of participants who received setrusumab 2, 8, 20 mg/kg, and 20 mg/kg open label, respectively, and infusion-related reactions were reported in 3.3%, 3.4%, 12.9%, and 4.8% of participants, respectively. All reported infusion-related reactions were mild in severity except one moderate reaction in the setrusumab 20 mg/kg group.

**Table 3:** Safety summary during the asteroid treatment period.

	**Setrusumab dose**			
**Participants, *n* (%)**	**2 mg/kg** ***n* = 30**	**8 mg/kg** ***n* = 29**	**20 mg/kg** ***n* = 31**	**20 mg/kg open label** ***n* = 21**
**Treatment emergent adverse event (TEAE)**	27 (90.0%)	26 (89.7%)	31 (100%)	20 (95%)
**Related TEAEs**	11 (36.7%)	12 (41.4%)	21 (67.7%)	9 (42.9%)
**TEAEs leading to study discontinuation^a^**	0	0	2 (6.5%)^a^	2 (9.5%)^b^
**Serious TEAEs**	4 (13.3%)	7 (24.1%)	4 (12.9%)	5 (23.8%)
**Related Serious TEAEs**	0	0	2 (6.5%)	2 (9.5%)
**Infusion site reactions**	1 (3.3%)	3 (10.3%)	5 (16.1%)	3 (14.3%)
**Infusion related reaction^c^**	1 (3.3%)	3 (10.3%)	4 (12.9%)	1 (4.8%)
**Injection site extravasation**	0	1 (3.4%)	1 (3.2%)	1 (4.8%)
**ADA^d^**	5 (16.7%)	5 (17.2%)	5 (16.1%)	2 (9.5%)

a Neutropenia and headache.

b Headache and worsening platybasia-related symptoms.

c All reactions were mild with the exception of one moderate infusion related reaction in the 20 mg/kg group.

d Includes month 14 assessment.

Abbreviations: ADA, antidrug antibodies; SAE, serious adverse events; TEAE, treatment emergent adverse event.

**Table 4:** Serious TEAEs reported during asteroid treatment period including unrelated events.

**Adverse event** ^^a^^ **, *n* (%)**	**2 mg/kg**	**8 mg/kg**	**20 mg/kg**	**20 mg/kg open label**
**Noninfective sialadenitis**	1 (3.3)	0	0	0
**Cholelithiasis**	0	1 (3.4)	0	0
**Anaphylaxis^b,c^**	0	0	1 (3.2)	0
**Appendicitis**	0	1 (3.4)	0	0
**Device-related infection**	0	0	0	1 (4.8)
**Joint abscess**	0	0	0	1 (4.8)
**Pulmonary hypertension**	0	0	0	1 (4.8)
**Worsening platybasia-related symptoms**	0	0	0	1 (4.8)
**Visual impairment**	0	0	0	1 (4.8)
**Chills**	0	0	0	1 (4.8)
**Wound infection**	0	1 (3.4)	0	0
**Arthralgia**	0	1 (3.4)	0	0
**Bone pain**	0	1 (3.4)	0	0
**Nonunion fracture**	1 (3.3)	0	0	0
**Headache^c^**	0	0	1 (3.2)	1 (4.8)
**Hydrocephalus^c^**	0	0	1 (3.2)	0
**Respiratory failure**	0	1 (3.4)	0	0

a Excluding fracture events.

b Initially reported as wheezing, urticaria, and pruritus, requiring ER treatment occurring approximately 60 hours after the fifth dose of setrusumab in a 65-yr-old female. The participant continued on study drug without any subsequent immune reaction. This event was considered by the investigator as possibly related to treatment and more likely related to an unspecified environmental or food allergen.

c Classified as related serious TEAEs.

Abbreviations: OI, osteogenesis imperfecta; TEAE, treatment emergent adverse event.

ADAs were detected during at least one visit of the treatment period in 16.7%, 17.2%, 16.1%, and 9.5% of participants in the setrusumab 2, 8, 20 mg/kg, and 20 mg/kg open label groups, respectively; 5 of these participants (1 per blinded group; 2 in the 20 mg/kg open label group) were positive for ADAs at baseline (pretreatment). Notably, ADA titers were not boosted by setrusumab in these participants. The effects of setrusumab on aBMD and serum P1NP did not differ according to ADA status, and serum setrusumab concentrations appeared unaffected by the presence of ADAs.

## Discussion

The phase 2b Asteroid study examined the impact of 12 mo of setrusumab treatment in adults with OI types I, III, and IV. During the treatment period, participants experienced significant improvements in bone health including microFE-derived measures of bone strength and areal BMD, but not the primary outcome of trabecular vBMD at the radius. On average, the 20 mg/kg dose resulted in the greatest improvements in bone endpoints, including vBMD increases, with no differences between type of OI.

HR-pQCT has been previously used to assess the trabecular and cortical bone density and microstructure of the nondominant distal radius and tibia in patients with OI.^17-20^ In a study conducted by Folkestad et al., adults with OI type I had lower trabecular vBMD, bone area, and trabecular number compared with healthy controls.^17^ In Asteroid, the primary endpoint examined the change from baseline in radial trabecular vBMD at month 12; however, this endpoint did not reach statistical significance. Although the mean baseline trabecular vBMD reported in Asteroid was similar to values reported by Folkestad and others, the range of baseline values observed in this study varied dramatically (−0.5 to 279.0 mg HA/cm^3^) and differed markedly from previously reported ranges.^17^ This corresponds to a coefficient of variation of 32%–42%, which is elevated compared with published levels of patients with OI (29%) and age- and sex-matched healthy controls (23%).^20^ The SD of all randomized setrusumab groups in Asteroid exceeded the expected value of 40 mg HA/cm^3^, further suggesting that an increased degree of baseline variability may have prevented the accurate measure of change in response to treatment. Because the sample size calculation was based on the expected variability of 40 mg HA/cm^3^, which was exceeded in the present population, it is likely that the analysis of vBMD was underpowered. Additionally, the mean baseline radial trabecular vBMD of participants in Asteroid was markedly low (110.2–127.1 mg HA/cm^3^) compared with published levels for healthy adults without OI (mean [SD] 190^10^ mg HA/cm^3^).^35^ Reported negative values of trabecular vBMD in Asteroid indicate that the mean density measured by HR-pQCT for the trabecular compartment, delineated by the endosteum, was likely dominated by fatty marrow (density < 0 mg HA/cm^3^) rather than bone, as reported in other studies.^40^ Anabolic agents such as setrusumab require bone surfaces on which new bone formation can occur; in many of these adult OI participants, trabecular bone was absent, limiting the potential efficacy of setrusumab. Together, the abnormally low baseline vBMD levels and high degree of variability in participants suggest that the trabecular compartment of these participants is variably deteriorated or insufficiently developed, making it an inconsistent region from which to examine drug-related changes thereby contributing to the observed failure to demonstrate an effect of setrusumab.

While the primary endpoint was not met, the clinically relevant endpoint of estimated bone strength and key secondary endpoints largely support the efficacy of setrusumab in improving bone health and quality. Notably, few approved therapeutic agents for postmenopausal osteoporosis that reduce nonvertebral fractures have been shown to also significantly increase radial vBMD from baseline. Thus, the findings from Asteroid presented herein are encouraging for a population with a lifelong increased fracture risk, as caused by an underlying defect in patients with OI, who have a significantly increased risk of long bone fractures.^41^

Improvements in the cortical and total vBMD of the tibia were observed with setrusumab, with higher doses corresponding to greater improvements, reaching statistical significance in the 20 mg/kg group. These improvements in total vBMD at the radius and tibia corresponded to increased bone stiffness and failure load by microFE, with higher doses corresponding to greater improvements. Of note, studies have shown that failure load is the best predictor of fracture risk among HR-pQCT parameters, further emphasizing the importance of this endpoint in Asteroid.^36^ Finally, cortical area of both the radius and tibia, and cortical thickness of the radius improved significantly from baseline at month 12 in the 8 and 20 mg/kg setrusumab groups.

Dose-responsive improvements in DXA aBMD of the LS, TH, FN, and total body were also observed with setrusumab treatment. At the LS, significant increases in TBS suggested improvements in trabecular microarchitecture with setrusumab. Improvements in aBMD were continuous across the 12-mo treatment period in several different bone regions, with similar changes observed from months 1-6 and months 7-12. This suggests that setrusumab maintained a positive balance of bone formation and resorption over at least a 12-mo treatment period. The mean percent change from baseline at 12 mo in aBMD in the 20 mg/kg dose group (9.0%) is markedly higher than what has been previously reported for other therapies in adults with OI.^22^^,^^42^ Orwoll et al. reported 4.7% in LS aBMD at 12 mo in adults with OI receiving teriparatide.^22^

The changes in BMD and bone strength in Asteroid were further supported by the changes in biomarkers of bone turnover. Bone formation markers (P1NP, OC, and BSAP) showed robust increases from baseline corresponding with setrusumab initiation, peaking at around month 1 before tapering through the end of the treatment period. Generally, higher setrusumab doses corresponded to greater improvements. Meanwhile, CTx1 showed a decrease by month 1 followed by a gradual return toward baseline through month 12. The observed peak effect at month 1 across biomarkers assessed is presumed to be due to increased bone modeling stemming from activation of quiescent bone surfaces and recruitment of progenitor cells. The antiresorptive action provided by setrusumab treatment results in a large spike in Wnt signaling and osteoblast differentiation before innate negative feedback loops activate, leading to the observed taper in biomarker levels.^43^^,^^44^ Similar increased bone formation and decreased bone resorption were observed in the prior phase 2a study of setrusumab in adults with OI,^33^ and tapering biomarker responses are consistent with clinical data generated for other anti-Scl-Ab in postmenopausal osteoporosis.^45^^,^^46^ Correlation analyses in the present study showed that these initial changes in bone biomarkers at month 1 were related to changes in LS aBMD following 12 mo of setrusumab. Similar trends have been described in studies of osteoporosis, wherein women treated with teriparatide showed early increases in biomarkers of bone formation related to 2-yr gains in BMD.^47^ Although these correlations using individual data are not particularly strong, the consistent correspondence between early increases in bone formation markers and later increases in BMD at the group level supports the utility of such biomarkers in gauging response to different dose levels of setrusumab in patients with OI.

Given the complex heterogenic nature of OI, it is important to consider the efficacy of setrusumab across all patients. This distinction is important, as OI types can have differing underlying pathologies and may respond differently to treatment.^2^^,^^4^ For example, while studies have shown that adults with OI have lower serum sclerostin levels vs age- and sex-matched controls (*p*<.0001), mean serum sclerostin levels in individuals with OI type III/IV were observed to be lower vs type I, although this outcome was not statistically significant (*p*=.14).^27^ While Asteroid was not designed to measure efficacy at the individual variant level, it is possible to infer these outcomes from assessments by OI type, as OI type I is associated with quantitative variants, and OI types III/IV with qualitative variants.^5^ Notably, the increases in aBMD and radius failure load, and changes in markers of bone formation and resorption observed in Asteroid were consistent across OI types analyzed (I vs III/IV). This suggests that setrusumab can provide benefit to a greater number of patients with OI, in contrast to other therapies such as teriparatide, which demonstrated efficacy only in OI type I.^22^

Together, setrusumab demonstrated efficacy in patients with OI in Asteroid and holds promise as a treatment option. Setrusumab and existing bisphosphonate therapies demonstrate BMD improvements in adults^10^; however, while the results of Asteroid are suggestive that setrusumab may reduce fracture rates in patients with OI, the effects of bisphosphonates remain unclear.^18^^,^^48^ It is purported that this is due to the mechanism of action of bisphosphonates in increasing bone mineralization, potentially also increasing the fragility of bones.^10^ In contrast, setrusumab reduces bone resorption through a biological, rather than a chemical, mechanism and recruits additional osteoblasts to enhance bone production naturally at sites of bone tension and fragility. This combination of increased cells and bone production with moderated resorption enhances the normal bone dynamic balance, distinguishing it from currently available therapies.

The most commonly reported related TEAEs during the treatment period were arthralgia, headache, and infusion-related reactions. One serious event of chills and headache on day 1 was reported as likely related to setrusumab. One anaphylactic reaction was believed to be possibly related to setrusumab treatment by investigators; this participant continued treatment without further symptoms after all subsequent doses. While infusion-related reactions were reported in 8 participants (9%), the assessment of causality was confounded by a site-specific issue related to IV placement. Importantly, no adverse events of neurologic impingement were reported in this study. While 3 reports of decreased blood calcium levels were observed, all changes were determined to be nonserious by investigators, and none resulted in study discontinuation. Moreover, no serious TEAE appeared in more than one patient during the Asteroid treatment period. Given the concern of cardiovascular effects following sclerostin inhibition, cardiovascular outcomes were included as adverse events of special interest and assessed during physical examinations (brief examinations monthly during the treatment period and every 4 mo during follow-up; complete examinations every 6 mo during the treatment period) and electrocardiogram (every 6 mo during the treatment period and 60 d posttreatment). Overall, the safety data were consistent with the anticipated safety profile of setrusumab, and no new treatment-associated safety concerns were identified, including cardiovascular events.

This study was limited by the lack of a placebo comparator group. Nevertheless, otherwise healthy adults with OI are expected to have a relatively stable skeletal status over a 12-mo period, and the evaluation of significant changes from baseline in Asteroid is likely appropriate. The study was also limited in its ability to determine the effect of setrusumab on fracture rate. Although a reduction in fracture rates is suggestive in the 20 mg/kg subgroup, the study was not powered to detect a significant effect. Larger and/or longer studies or studies in patients with OI who have higher predicted fracture rates are required to overcome potentially confounding aspects of fracture rate detection and demonstrate a significant reduction. Here, it is important to consider the impact of posttreatment behavior on fracture susceptibility. As subjects undergo treatment, potential reductions in fatigue and pain coupled with improved confidence in treatment may lead to increases in physical activity, thereby increasing fracture risk. Without patient-reported quality of life and activity outcome measures, it is difficult to assess this impact on fracture risk. These elements were incorporated into the design of the ongoing pivotal study of the effect of setrusumab on fracture risk in patients with OI (NCT05125809).

In the present study, setrusumab treatment resulted in significant improvements in a number of measures of bone health including microFE-derived measures of bone strength and aBMD. Critically, these improvements with setrusumab were consistent across OI type, supporting its treatment potential across disease severity. The 20 mg/kg dose resulted in the greatest improvements in bone endpoints, including BMD increases that were sustained through the 12-mo treatment period. The safety data for setrusumab were consistent with the expected safety profile, and no new treatment-associated safety concerns were identified, including no cardiovascular events. Together, these positive outcomes of setrusumab in adults with OI have led to the preparation for phase 3 evaluation.

## Supplementary Material

ASTEROID_Manuscript_Revisions_Supplement_v3_0_31May2024_CLEAN_zjae112

## Data Availability

The data underlying this article will be shared on reasonable request to the corresponding author.
