# Correction to: Setrusumab for the treatment of osteogenesis imperfecta: 12-month results from the phase 2b asteroid study

**DOI:** 10.1093/jbmr/zjaf031

**Published:** 2025-03-03

This is a correction to: Francis H Glorieux et al., Setrusumab for the treatment of osteogenesis imperfecta: 12-month results from the phase 2b asteroid study, *Journal of Bone and Mineral Research*, Volume 39, Issue 9, September 2024, Pages 1215–1228, https://doi.org/10.1093/jbmr/zjae112

In the version originally published, the x-axis in Figure 1B was misplaced, passing through the value of `4' on the y-axis. The figure has been corrected for clarity.

