# Supplementary material for: Setrusumab for the treatment of osteogenesis imperfecta: 12-month results from the phase 2b asteroid study
Source: J Bone Miner Res. 2024 Jul 16;39(9):1215–28. doi: 10.1093/jbmr/zjae112 (PMC11371902; doi:10.1093/jbmr/zjae112)
Supplement: ASTEROID_Manuscript_Revisions_Supplement_v3_0_31May2024_CLEAN_zjae112 [file asteroid_manuscript_revisions_supplement_v3_0_31may2024_clean_zjae112.docx]

**Supplementary Materials**

**Methods**

**Supplementary Figure 1. Asteroid Study Design**


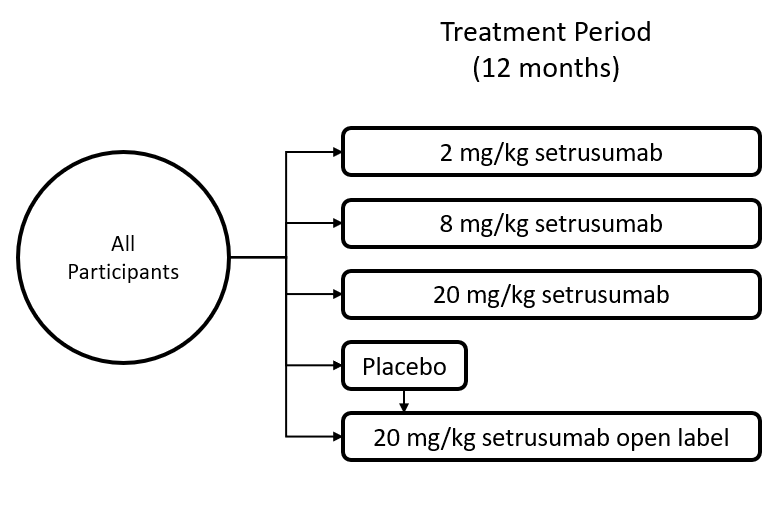


**HR-pQCT**

Measures of limb length were taken by the same staff at each site to ensure consistency. The XtremeCT and XtremeCT II scans contained 110 slices at 82 µm or 168 slices at 60.7 µm isotropic voxel size, respectively. At each imaging site, a trained technician graded the scout view scan and repeated a scan (up to a total of 3 scans) if motion grade was 4 or higher (1). Images were sent to the central HR-pQCT reader who independently graded the images and excluded images with excessive motion artifact (score ≥4). Periosteal contouring was performed at each HR-pQCT scanning center, with contouring confirmed by the central HR-pQCT reader prior to analysis using the manufacturer’s standard evaluation protocol (Scanco Medical AG). For CSA registration, an overlapping volume criterion was not implemented to omit participant data, as the presence of multiple timepoints resulted in an overlap of less than 80% for 23% (radius) and 12% (tibia) of the participant scans with at least two timepoints. However, only 6% (radius) and 4% (tibia) of the scans had less than 70% overlap. A reference line was positioned at the medial proximal margin of the radial articular surface or at the tibial plateau (2). The scan occurred over a volume of interest centered at a distance of 4% (radius) and 7% (tibia) of the ulna or tibial length from the reference line (2, 3).

All sites performed periodic quality control HR-pQCT imaging throughout the study using s common quality control phantom (QC1, Scanco Medical AG) and European Forearm Phantom (EFP, QRM GmbH). The methodology used to perform cross-calibration across sites and generate precision errors was previously described (4, 5). The *in vivo* root-mean-squared short-term precision error (CV%_rms_) from participants ranged between 1.3%–3.8% (radius) and 0.9%–1.9% (tibia) for density and area measurements. Due to delays in circulating phantoms between scanning centers, participant data was calibrated using the most recent phantom scan available, rather than matching participant time points to phantom timepoints. In cases where no calibration data were available (13.8% of scans), the uncalibrated participant data were retained and used as-is. The retention of these uncalibrated values was justified based on comparisons of pre- and post-calibrated values; the differences between pre- and post-calibrated values were not significant (p > 0.05 by paired t-test). Furthermore, R^2^ values ranged between 0.988 to 1, and mean percentage differences ranged between -1.85% to 1.97%. For the rare cases when participants were not scanned on the same scanner as at baseline, 6, and 12 months, during cross-calibration, the different scanners were accounted for regarding these participants’ scans.

**Micro Finite Element Analysis**

Following the analysis of the density and microarchitectural outcomes, the VOI identified using CSA registration was used by the central HR-pQCT lab to simulate axial compression (IPLFE v01.16, Scanco Medical AG). A linear elastic modulus of E=6829 Mpa, and E=8748 Mpa was applied for XCT and XCT2 scans, respectively (6, 7, 8) and a Poisson’s ratio was set to 0.3. The CV%_rms_ for microFE outcomes ranged between 0.7%–3.0% as reported elsewhere (4, 5).

To estimate bone stiffness and strength in Asteroid, microFE analyses incorporated the tissue-independent Pistoia’s criterion. A Pistoia’s failure criteria with critical volume of 2% and critical value of 7000 µstrain was used (9, 10). This failure criterion has been validated in healthy bones and is applied here in individuals with OI. MicroFE accounts for altered microarchitecture of OI bone, but not for potential changes in the material properties at the microscopic scale, which are still poorly understood. Furthermore, setrusumab treatment is not expected to change the material-level properties between baseline and follow-up, consistent with bone biopsy data from the Asteroid trial that did not find differences in bone matrix or mineral characteristics between non-treated OI and setrusumab-treated bone (Rummler et al., *JBMR*; in review).

**DXA**

Phantom measurements were collected and analyzed each day a study subject was scanned, but not less than three days a week per scanner. Change in scanner calibration from the baseline calibration was assessed using CUSUM analysis. The change in scanner calibration at each identified breakpoint was evaluated for both statistical and clinical significance. Statistical significance was defined as a p-value less than 0.05 for a t-test of mean phantom BMD before and after the break point. Clinical significance was a difference in BMD across the breakpoint greater than 0.5%. Nonsignificant breakpoints were considered normal variation within scanner specifications and are ignored. Significant breakpoints represent unacceptable changes in calibration and warrant correction.

**Analysis of Bone Turnover Biomarkers**

Serum samples were allowed to clot for a minimum of 30 minutes at room temperature before being centrifuged (2000 g for 15 minutes at +4°C or room temperature) no later than 1 hour after sampling. Serum was collected and frozen at -20°C (for up to 2 weeks) or -80°C before shipping to a central laboratory for analysis. P1NP was measured using the Elecsys® total P1NP electrochemiluminescence sandwich-based immunoassay with a lower limit of quantification (LLOQ) of 8.99 ng/mL; OC was measured using the Elecsys® N-mid OC electrochemiluminescence sandwich-based immunoassay with an LLOQ of 6.54 ng/mL; and CTx1 was measured using the Elecsys® beta-CrossLaps electroluminescence assay with an LLOQ of 0.082 ng/mL (all assays from Roche Diagnostics, Mannheim, Germany). BSAP enzymatic activity was measured using the MicroVue BSAP enzyme immunoassay with a LLOQ of 4.95 U/L (both assays from Quidel Corporation, San Diego, CA, USA).

Statistical Analysis

*Sample Size Determination*

The target sample size was based on power considerations for the trabecular vBMD derived from HR-pQCT at Month 12. Under the assumptions of a maximum change Baseline to Month 12 of 20 mg HA/cm³, a standard deviation at both time points of 40 mg HA/cm³, and a Pearson correlation between both measurements of 0.45, a sample size of 25 subjects per group yields approximately 80% power for a 1-sided test at a significance level of 0.025 if the data are analyzed on the log-scale using a t-test.

*Statistical Evaluation*

The statistical evaluation was performed using SAS®, Version 9.4 or higher and ADDPLAN DF 4.0 or later. Continuous variables were summarized using descriptive statistics including number of non‑missing observations (n), means, and standard error (SEM). For categorical variables, summaries included the number of non‑missing observations (n) or the number of participants in the population (N) and as applicable, the counts of participants and percentages.

For continuous outcomes, an analysis of covariance (ANCOVA) model was used, including the fixed categorical effects of treatment and randomization stratum, and associated baseline values as covariates.

Least square (LS) Means and LSMean differences between groups were back-transformed to their original scale for descriptive purposes. After subtracting the geometric mean of the log baseline values, the back-transformed LSMeans correspond to the percentage change from baseline, while the back-transformed LSMeans differences correspond to the ratio of percentage changes from baseline.

Count variables (e.g., number of new fractures) were analyzed using Poisson regression. Analyses included treatment and randomization as fixed effects and the logarithm of the duration of exposure to study medication was included as an offset variable in the model. For binary outcomes, the proportion of participants with a response was summarized and compared between treatment groups using Chi-squared test.

Statistical testing was two-sided and conducted at the significance (alpha) level of 0.05. Two-sided 95% confidence intervals (CIs) are provided when relevant. For the primary analysis, the LSMeans for each blinded treatment group will be calculated and tested against the null hypothesis of 0 in an a priori hierarchical approach, starting with the highest dose. For secondary and exploratory endpoints treatment groups will be assessed by pairwise comparisons for secondary efficacy data, and no adjustment for multiplicity will be made.

Pearson correlations were calculated between changes from baseline in Month 1 biomarkers of bone turnover and percent change from baseline in Month 12 aBMD to examine the predictive value of early biomarker responses for long-term changes in bone density.

**Supplementary References**

1. Pialat JB, Burghardt AJ, Sode M, Link TM, Majumdar S. Visual grading of motion induced image degradation in high resolution peripheral computed tomography: impact of image quality on measures of bone density and micro-architecture. Bone. 2012;50(1):111-8.
2. Bonaretti S, Majumdar S, Lang TF, Khosla S, Burghardt AJ. The comparability of HR-pQCT bone measurements is improved by scanning anatomically standardized regions. Osteoporos Int. 2017;28(7):2115-28.
3. Whittier DE, Boyd SK, Burghardt AJ, Paccou J, Ghasem-Zadeh A, Chapurlat R, et al. Guidelines for the assessment of bone density and microarchitecture in vivo using high-resolution peripheral quantitative computed tomography. Osteoporos Int. 2020;31(9):1607-27.
4. Mikolajewicz N, Zimmermann EA, Rummler M, Hosseinitabatabaei S, Julien C, Glorieux FH, et al. Multisite longitudinal calibration of HR-pQCT scanners and precision in osteogenesis imperfecta. Bone. 2021;147:115880.
5. Hosseinitabatabaei S, Mikolajewicz N, Zimmermann EA, Rummler M, Steyn B, Julien C, et al. 3D image registration marginally improves the precision of HR-pQCT measurements compared to cross-sectional-area registration in adults with osteogenesis imperfecta. J Bone Miner Res. 2022.
6. Macneil JA, Boyd SK. Bone strength at the distal radius can be estimated from high-resolution peripheral quantitative computed tomography and the finite element method. Bone. 2008;42(6):1203-13.
7. van Rietbergen B, Ito K. A survey of micro-finite element analysis for clinical assessment of bone strength: the first decade. J Biomech. 2015;48(5):832-41.
8. Whittier DE, Manske SL, Kiel DP, Bouxsein M, Boyd SK. Harmonizing finite element modelling for non-invasive strength estimation by high-resolution peripheral quantitative computed tomography. J Biomech. 2018;80:63-71.
9. Pistoia W, van Rietbergen B, Lochmüller EM, Lill CA, Eckstein F, Rüegsegger P. Estimation of Distal Radius Failure Load With Micro-Finite Element Analysis Models Based on Three-dimensional Peripheral Quantitative Computed Tomography Images. Bone. 2002;30(6):842–8.
10. Hosseinitabatabaei S, Kawalilak CE, McDonald MP, Kontulainen SA, Johnston JD. Distal radius sections offer accurate and precise estimates of forearm fracture load. Clin Biomech (Bristol, Avon). 2020;80:105144.
11. Indermaur M, Casari D, Kochetkova T, Peruzzi C, Zimmermann E, Rauch F, et al. Compressive Strength of Iliac Bone ECM Is Not Reduced in Osteogenesis Imperfecta and Increases With Mineralization. J Bone Miner Res. 2021;36(7):1364-75.
12. Indermaur M, Casari D, Kochetkova T, Willie BM, Michler J, Schwiedrzik J, et al. Tensile Mechanical Properties of Dry Cortical Bone Extracellular Matrix: A Comparison Among Two Osteogenesis Imperfecta and One Healthy Control Iliac Crest Biopsies. JBMR Plus. 2023;7(12):e10826.
13. (Rummler et al., *JBMR*; in review)

**Results**

The co-primary and key secondary endpoints of the setrusumab 20 mg/kg OL were generally comparable to those of the randomized setrusumab groups and are reported in Supplementary Table 1 below.

**Supplementary Table 1:** Co-Primary and Key Secondary Outcomes From the Setrusumab 20 mg/kg Open Label group

| **Mean (SEM) Change from Baseline at Month 12** | **Setrusumab 20 mg/kg OL** |
| --- | --- |
| *Co-Primary Endpoints* | |
| Radial Trabecular vBMD, %  n | 1.30 (1.6043)  16 |
| Radial Stiffness, N/mm  n | 4911.83 (1452.7)  15 |
| Radial Failure Load, N  n | 93.97 (32.6)  15 |
| *Key Secondary Endpoints* | |
| Radial Cortical vBMD  n | 1.09 (0.78)  16 |
| Radial Total vBMD  n | 2.33 (1.91)  16 |
| Tibial Trabecular vBMD  n | 2.46 (0.91)  15 |
| Tibial Cortical vBMD  n | 0.81 (0.79)  15 |
| Tibial Total vBMD  n | 1.93 (0.97)  15 |
| Tibial Stiffness, N/mm  n | 5452.62 (2490.22)  15 |
| Tibial Failure Load, N  n | 105.38 (42.40)  15 |
| Lumbar Spine aBMD, %  n | 9.38 (1.47)  15 |
| Total Hip aBMD, %  n | 3.03 (0.82)  14 |
| Femoral Neck aBMD, %  n | 4.14 (1.38)  14 |
| Total Body Excluding Head aBMD, %  n | 3.03 (0.84)  18 |

aBMD, areal bone mineral density; OL, open label; SEM, standard error of the mean; vBMD, volumetric bone mineral density.

**Supplementary Table 2:** Mean (SD) Baseline DXA T-scores and Z-scores

| **Setrusumab Dose** | **2 mg/kg** | **8 mg/kg** | **20 mg/kg** |
| --- | --- | --- | --- |
| Lumbar Spine T-score | -2.4 (1.3) | -2.3 (1.1) | -2.6 (1.4) |
| Lumbar Spine Z-score | -1.7 (1.4) | -1.9 (1.1) | -2.1 (1.6) |
| Total Hip T-score | -1.9 (1.2) | -1.5 (1.1) | -1.6 (1.1) |
| Total Hip Z-score | -1.4 (1.0) | -1.1 (1.0) | -1.3 (1.0) |
| Femoral Neck T-score | -1.9 (1.0) | -1.7 (0.9) | -1.7 (1.1) |
| Femoral Neck Z-score | -1.1 (0.8) | -1.1 (0.7) | -1.1 (0.9) |

DXA, dual X-day absorptiometry.

**Supplementary Table 3:** Absolute Change in microFE From Baseline After 12 Months of Setrusumab

| **Setrusumab Dose** | **2 mg/kg** | **8 mg/kg** | **20 mg/kg** |
| --- | --- | --- | --- |
| **LS Mean (SE) Change** |  |  |  |
| Radius Failure Load | 11.0 (30.1) N | 32.2 (24.6) N | 63.5 (22.0) N |
| Radius Stiffness | 209.9 (671.8) N/mm | 1422.0 (703.3) N/mm | 1638.7 (625.8) N/mm |
| Tibia Failure Load | 62.3 (51.8) N | 65.8 (48.6) N | 82.6 (41.2) N |
| Tibia Stiffness | 1428.9 (1339.9) N/mm | 1543.9 (1254.0) N/mm | 2326.6 (1048.5) N/mm |

microFE, micro finite element analysis.

**Supplementary Table 4:** Baseline and Month 12 values for HR-pQCT measurements at the distal radius and Tibia

|  | **Baseline Value, mean (SD)** | | | **Month 12, mean (SD)** | | |
| --- | --- | --- | --- | --- | --- | --- |
| **Setrusumab Dose** | **2 mg/kg**  **n=30** | **8 mg/kg**  **n=29** | **20 mg/kg**  **n=31** | **2 mg/kg**  **n=30** | **8 mg/kg**  **n=29** | **20 mg/kg**  **n=31** |
| Trabecular Bone Volume Fraction (Radius), mm^3^/mm^3^ | 0.15 (0.05) | 0.14 (0.06) | 0.17 (0.07) | 0.14 (0.06) | 0.14 (0.05) | 0.16 (0.05) |
| Trabecular Bone Volume Fraction (Tibia), mm^3^/mm^3^ | 0.13 (0.06) | 0.13 (0.07) | 0.13 (0.10) | 0.12 (0.07) | 0.12 (0.07) | 0.11 (0.05) |
| Cortical Area (Radius), mm^2^ | 47.30 (10.30) | 51.58 (9.88) | 48.63 (7.14) | 47.76 (10.57) | 54.24 (10.70) | 49.93 (8.26) |
| Cortical Area (Tibia), mm^2^ | 107.75 (32.62) | 100.03 (29.72) | 76.54 (32.90) | 109.67 (31.12) | 102.60 (30.41) | 79.50 (31.31) |
| Cortical Thickness (Radius), mm | 0.62 (0.10) | 0.74 (0.14) | 0.71 (0.14) | 0.63 (0.10) | 0.76 (0.16) | 0.73 (0.16) |
| Cortical Thickness (Tibia), mm | 1.12 (0.40) | 1.02 (0.33) | 0.81 (0.31) | 1.14 (0.38) | 1.04 (0.34) | 0.83 (0.29) |

HR-pQCT, high resolution peripheral quantitative computed tomography.

**Supplementary Figure 2:** Representative distal (top row), middle (middle row) and proximal (bottom row) slices of the distal radius scan with negative Tb.vBMD at 3 timepoints (columns). The images indicating the lack of trabeculae resulting in the negative values. The green lines indicate the periosteal and endosteal contours.


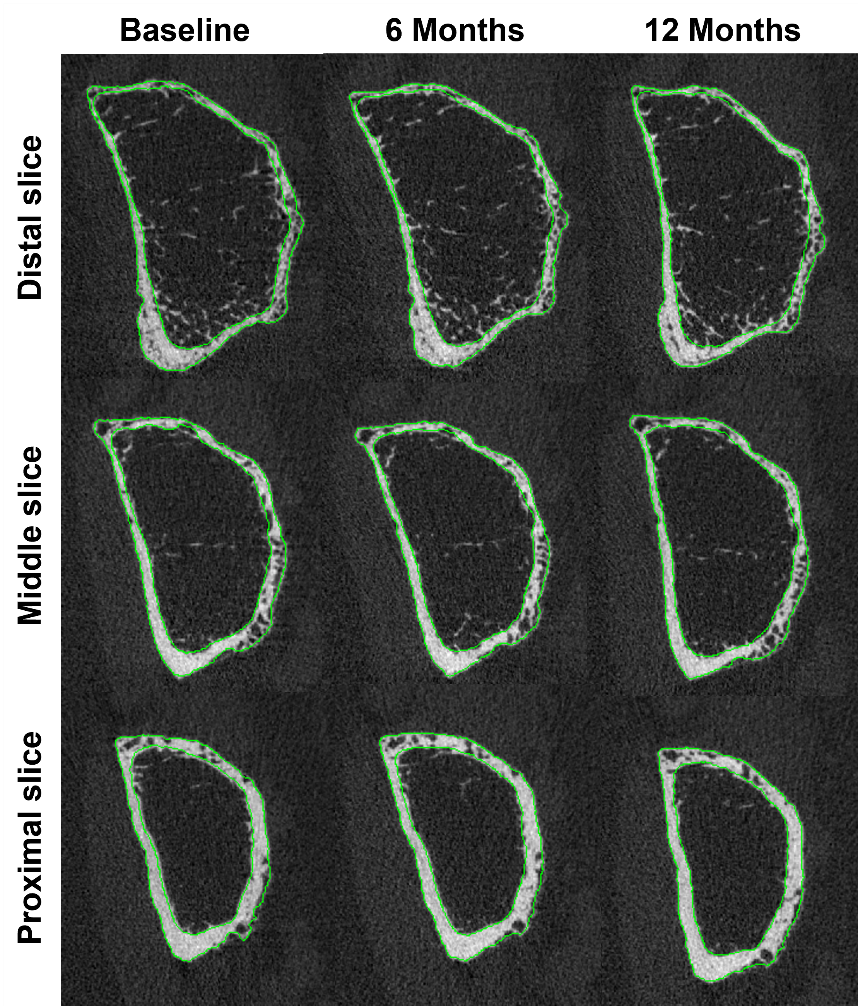


Tb.vBMD, trabecular volumetric bone mineral density.

**Supplementary Figure 3:** Mean (SEM) Change from Baseline in Areal BMD by DXA of the lumbar spine (A), total hip (B), femoral neck (C), and total body excluding head (D)

**Supplementary Figure 3A: Lumbar Spine**

**
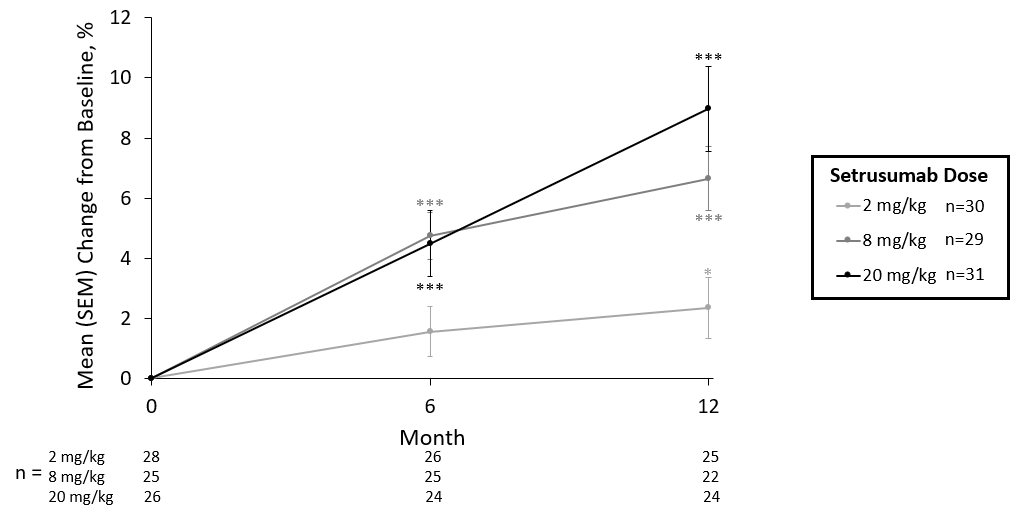
**

**Supplementary Figure 3B: Total Hip**

**
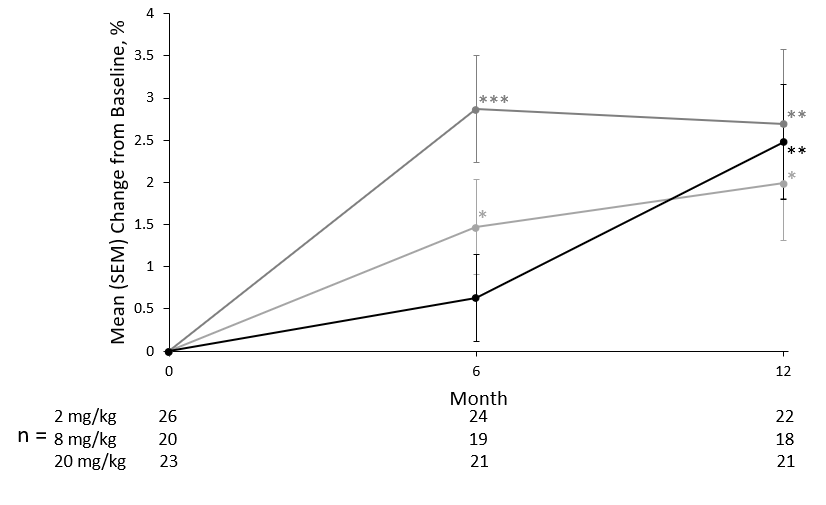
**

**Supplementary Figure 3C: Femoral Neck**

**
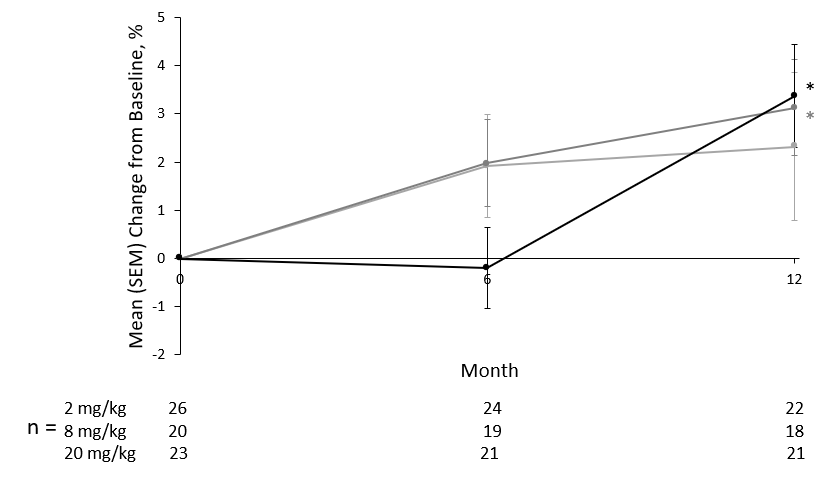
**

**Supplementary Figure 3D: Total Body Excluding Head**

**
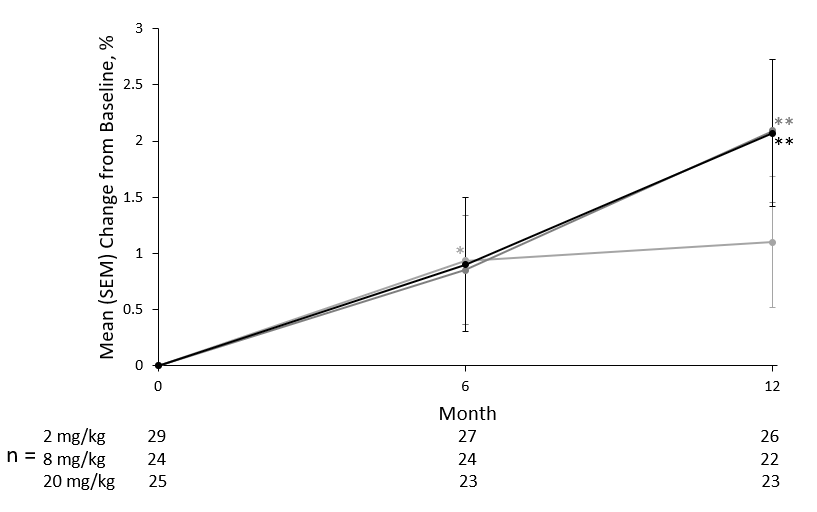
**

*p<0.05, **p<0.01, ***p<0.001 vs baseline based on an ANCOVA model with baseline values, treatment group and OI type as covariates

ANCOVA, analysis of covariance; BMD, bone mineral density; DXA, dual-energy x-ray absorptiometry; HR-pQCT, high resolution peripheral quantitative computed tomography; OI, osteogenesis imperfecta, M; month.

**Supplementary Figure 4. Correlation of Percent Change from Baseline in Serum P1NP (A) and BSAP (B) at Month 1 and Lumbar Spine aBMD at Month 12**

**Supplementary Figure 4A. P1NP**

**
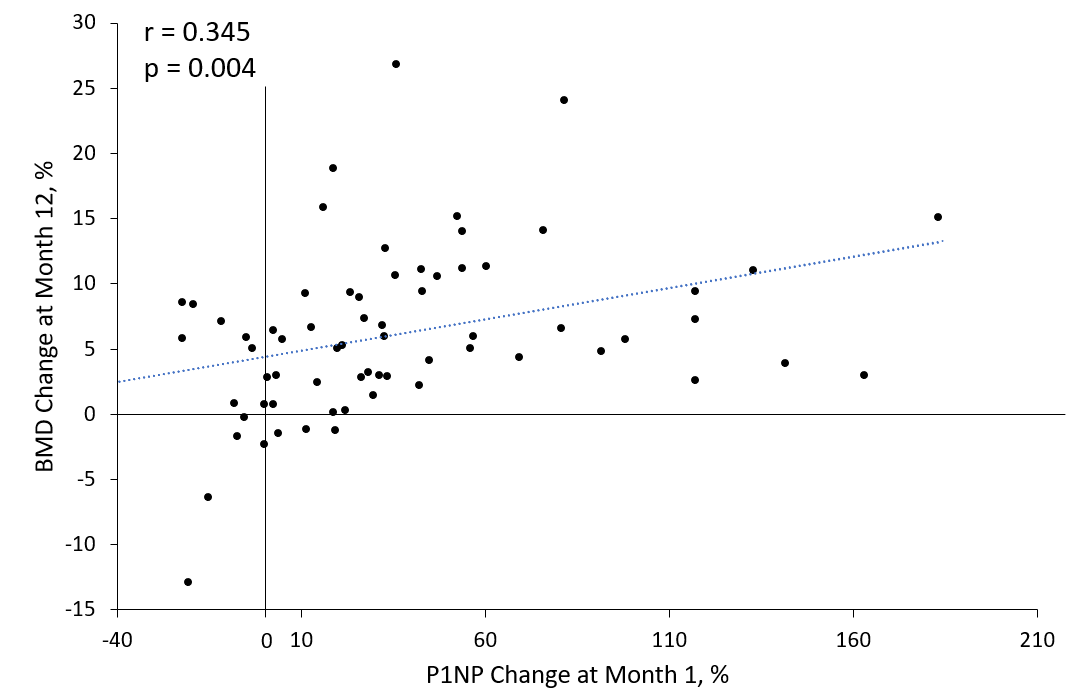
**

**Supplementary Figure 4B. BSAP**
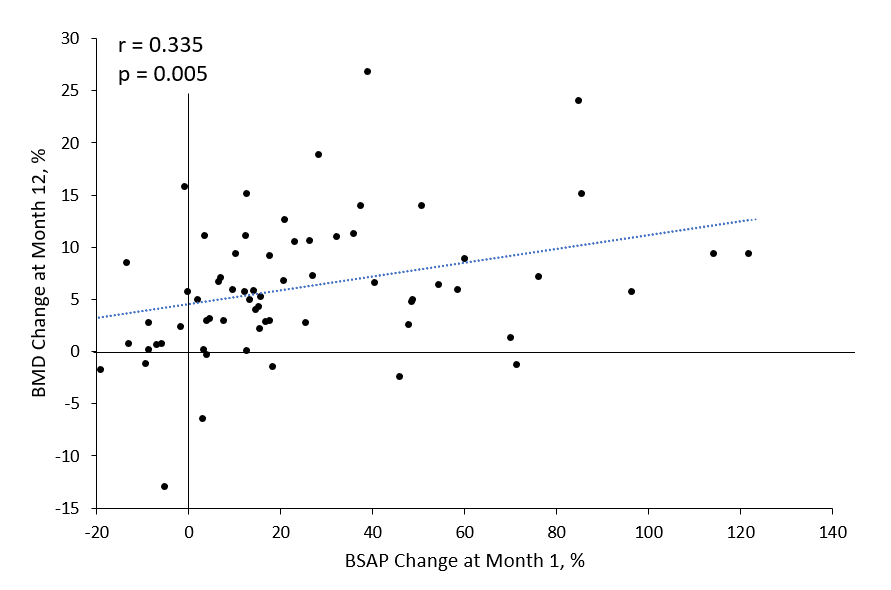


BSAP, bone-specific alkaline phosphatase; BMD, bone mineral density; P1NP, procollagen 1 intact-N-terminal propeptide.

**Supplementary Figure 5. Analysis of Key Endpoints by OI Type**

**Figure 5A. Percent Change from Baseline in Lumbar Spine aBMD at Month 12 by OI Type**


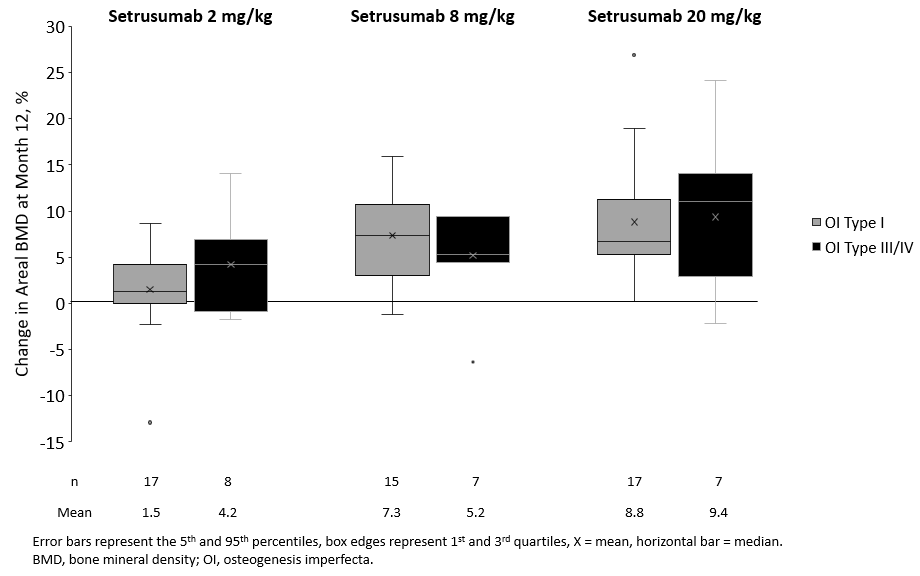


**Figure 5B. Percent Change from Baseline in Radius Failure Load at Month 12 by OI Type**


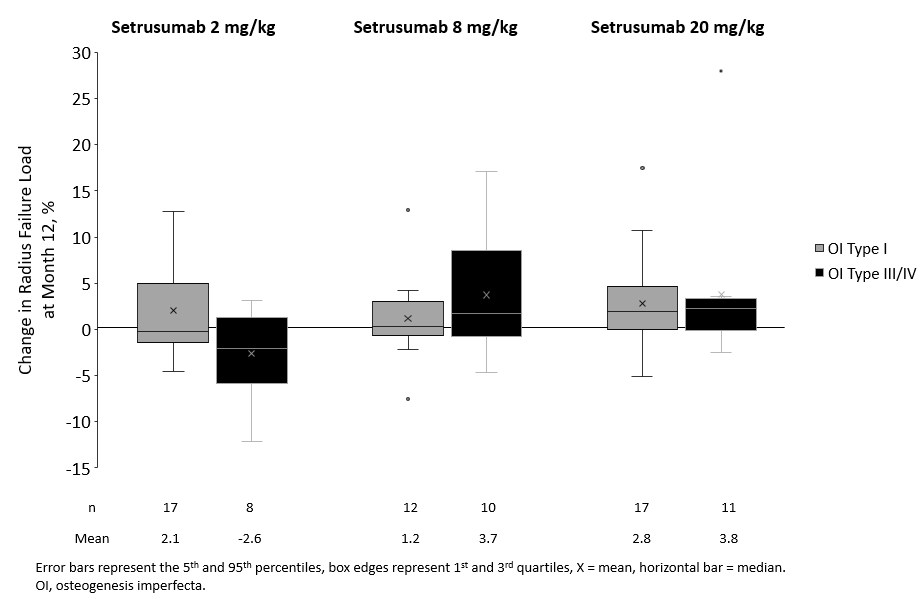


**Figure 5C. Percent Change from Baseline in Serum P1NP at Month 1 by OI Type**


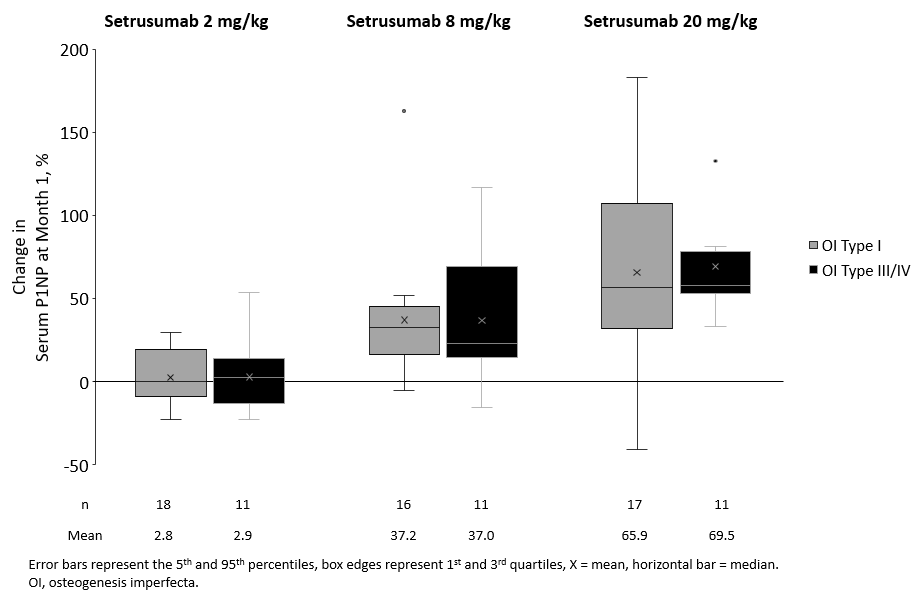


**Figure 5D. Percent Change** **from Baseline in Serum CTx1 at Month 1 by OI Type**


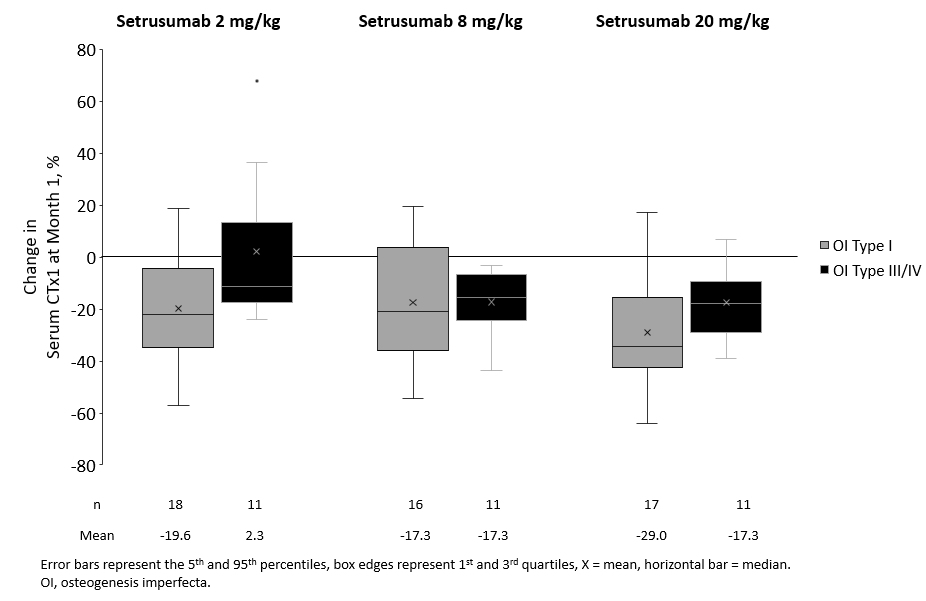


**Supplementary Table 5. Rate of Fracture During 12 Months of Setrusumab Treatment**

|  | **Setrusumab Dose** | | |
| --- | --- | --- | --- |
| **Fracture Location** | **2 mg/kg n=30** | **8 mg/kg n=29** | **20 mg/kg n=31** |
| Upper Limb   (radius, wrist, upper limb) | 1 | 1 | 1 |
| Lower Limb   (fibula, talus, tibia) | 0 | 3 | 0 |
| Pelvis | 0 | 0 | 1 |
| Rib | 5 | 3 | 1 |
| Hand and Foot | 3 | 5 | 2 |
| Long Bone (Unspecified) | 0 | 1 | 0 |
| Lumbar vertebral | 0 | 0 | 0 |
| Femur | 1 | 1 | 0 |
| Total Fractures | 10 | 14 | 5 |
| Number of Participants with Fractures | 7 (23.3%) | 10 (34.5%) | 5 (16.1%) |
| Annualized Fracture Rate  per Participant Year | 0.38 | 0.54 | 0.19 |
